# Reflecting on Earlier Experiences with Unsolicited Findings: Points to Consider for Next-Generation Sequencing and Informed Consent in Diagnostics

**DOI:** 10.1002/humu.22370

**Published:** 2013-07-16

**Authors:** Tessel Rigter, Lidewij Henneman, Ulf Kristoffersson, Alison Hall, Helger G Yntema, Pascal Borry, Holger Tönnies, Quinten Waisfisz, Mariet W Elting, Wybo J Dondorp, Martina C Cornel

**Affiliations:** 1 Department of Clinical Genetics, Section of Community Genetics and the EMGO Institute for Health and Care Research, VU University Medical CenterAmsterdam, The Netherlands; 2 Department of Clinical Genetics, University and Regional Laboratories, Region Skåne, and Lund UniversityLund, Sweden; 3 PHG FoundationCambridge, United Kingdom; 4 Department of Human Genetics, Radboud University Nijmegen Medical CentreNijmegen, The Netherlands; 5 Department of Public Health, Centre for Biomedical Ethics and LawLeuven, Belgium; 6 Robert Koch-Institut, Geschäftstelle Gendiagnostik-KommissionBerlin, Germany; 7 Department of Clinical Genetics, VU University Medical CenterAmsterdam, The Netherlands; 8 Department of Health, Ethics & Society, Research Schools CAPHRI and GROW, Maastricht UniversityMaastricht, The Netherlands

**Keywords:** high-throughput nucleotide sequencing, incidental findings, unsolicited findings, diagnosis, informed consent

## Abstract

High-throughput nucleotide sequencing (often referred to as next-generation sequencing; NGS) is increasingly being chosen as a diagnostic tool for cases of expected but unresolved genetic origin. When exploring a higher number of genetic variants, there is a higher chance of detecting unsolicited findings. The consequential increased need for decisions on disclosure of these unsolicited findings poses a challenge for the informed consent procedure. This article discusses the ethical and practical dilemmas encountered when contemplating informed consent for NGS in diagnostics from a multidisciplinary point of view. By exploring recent similar experiences with unsolicited findings in other settings, an attempt is made to describe what can be learned so far for implementing NGS in standard genetic diagnostics. The article concludes with a set of points to consider in order to guide decision-making on the extent of return of results in relation to the mode of informed consent. We hereby aim to provide a sound basis for developing guidelines for optimizing the informed consent procedure.

## Introduction

Advances in DNA sequencing techniques have intensified the use of sequencing large parts of the genome (next-generation sequencing, NGS) in research as well as in diagnostics. The challenge of NGS in clinical practice is to identify the pathogenic variants among the many thousands of (new) variants that could be detected in each genome [Majewski et al., 2011]. When analyzing the sequence for diagnostic purposes, the first step would preferably be a targeted approach, searching for pathogenic variants in a panel of known genes causing the clinical phenotype or in some cases for de novo mutations by comparing a child's genome with its parents [de Ligt et al., 2012]. When this first step does not answer the clinical enquiry, more regions of the sequence can be examined, increasing the chance of finding variants that lie outside of the clinical enquiry. These variants may or may not have clinical implications for the person himself and/or family members. In some cases, sequencing may thus be seen as a kind of screening, where screening should be understood as (predictive) testing without a specific indication [Hastings et al., 2012; van El et al., 2013].

A term frequently used for findings not related to the primary enquiry is “incidental finding” [Green et al., 2013]. However, since in large-scale genomics applications we expect findings other than the ones answering the clinical enquiry, these findings are not considered an incident. In this context, therefore, the term “unsolicited” seems more appropriate, as is also proposed by the European Society of Human Genetics [Hastings et al., 2012].

Before the advent of NGS, other existing diagnostic technologies within and outside genetics (including imaging and karyotyping) have existed with a low but significant chance of unsolicited findings [Berland et al., 2010; Kohane et al., 2012]. With NGS, the prospects for these findings are increased, requiring a re-examination of this topic [Johnston et al., 2012; Netzer et al., 2009; Ostrer, 2011]. Consequently, guidance is needed on what information should be discussed and consented to preceding the test; which results to disclose to the patient (which may have consequences for relatives) and the extent to which patients should decide on what should be reported back to them [Berg et al., 2011; Ormond et al., 2010; Ostrer, 2011; Thorogood et al., 2012]. Although the criteria for the informed consent procedure for NGS are currently a subject of debate within the professional community [Thorogood et al., 2012; Wright et al., 2011], no practical guidance has been described so far [Jackson et al., 2012].

To develop the best practice guidelines for an informed consent procedure for NGS in diagnostics, an international and multidisciplinary expert meeting entitled “Exome sequencing in diagnostics: exploring needs for the informed consent procedure” was organized in Amsterdam, The Netherlands (March 2012, Supp. Table S1). This article reflects the expert contributions and discussions of that meeting addressing specific questions, supported by additional literature searches. It aims to describe what can be learned so far and provides a set of questions that could be used to provide a sound basis for developing an optimal informed consent procedure for NGS in diagnostics. The following questions are addressed: (1) What are the ethical *challenges expected* in the first experiences with informed consent for NGS in diagnostics? (2) What are the ethical and practical *dilemmas encountered* in discussions on unsolicited findings and informed consent *so far* (from NGS and similar settings)? (3) What are the *practical needs and considerations* encountered in experiences with unsolicited findings and informed consent?

## Ethical Challenges Expected for Informed Consent for NGS in Diagnostics: About Patient Rights and Professional Duties

A patient's right to informed consent requires a balance between information overload and uninformed consent. As the range of possible outcomes of diagnostic NGS is generally too wide to be able to discuss and comprehend them all prior to testing, it has been proposed that a “generic consent” would be appropriate [Dondorp et al., 2012]. The extent to which “generic consent” is sufficiently informed and how much information is needed requires further study. These two aspects may well be context dependent.

When analyzing large parts of the genome diagnostically, a question arises on the patient's right to be informed about unsolicited findings. Informing patients about all unsolicited findings might not be practically possible due to time constraints; the cost of validating all findings; and the outcomes being of very low relevance such that informing patients might cause more harm than good (a conflict between nonmaleficence [avoiding the causation of harm] and autonomy).

In principle, patients also have a right not to be informed about unsolicited findings. This would mean that when being asked to give consent for a NGS-based test, patients should (within the limits of practicality) be given the opportunity to indicate both: which unsolicited findings they would want to be informed about and which not. The latter might lead to difficult situations where the physician is confronted with conflicting duties of respecting a patient's right not to know on the one hand and his/her duty to warn on the other.

Because patients (and physicians alike) might not be aware of all possible implications of potential findings before specific results have been generated, it seems appropriate to inform the patient about the fact that an absolute right not to know cannot be guaranteed. Another reason for this is that the interests of the patient's relatives may also be at stake [Thorogood et al., 2012]. In case of proxy consent (e.g., parents or legal representatives giving consent for their child), parents cannot claim a right not to know with regard to unsolicited findings that would be directly relevant for the child's health in the sense of requiring treatment, prevention, or surveillance. Furthermore, the right to know of parents is not absolute, since children also have a “right to an open future” [Bredenoord et al., 2013; Dondorp et al., 2012].

## Experiences with Unsolicited Findings and Informed Consent So Far

How to deal with unsolicited findings and the implications for informed consent has been discussed in different medical settings, for example, in the context of imaging [e.g., Mirilas and Skandalakis, 2002], neonatal screening [e.g., Miller et al., 2009], genetic research [e.g., Wolf et al., 2008], array comparative genomic hybridization (ArrayCGH) [e.g., Pichert et al., 2011], and NGS in diagnostics [e.g., Ormond et al., 2010]. A selection of experiences in these different settings is described here to explore the ethical and practical dilemmas encountered.

### Disclosing Carrier Status in Newborn Screening

Although newborn screening typically aims to identify newborns with severe disorders to start early treatment, as a result of the screening technology a heterozygous carrier status is (unsolicitedly) found in a relatively large proportion of children, for example, with regard to haemoglobinopathies. The detection of carriers and feedback of this information to parents has generated much discussion [Bombard et al., 2009; Miller et al., 2009]. Carrier status has no direct implications for the child being screened, but the information could be relevant in adult life, for example, with regard to reproductive choices. Because of additional potential relevance for family members and future reproductive decision making for the parents, it was decided in some countries that by default parents should be informed about this result. However, since it is not a goal of the screening, in some jurisdictions, parents can opt out for this finding, for example, in The Netherlands, by signing the bloodspot card (Supp. Fig. S1). It should be noted that the discussion about how to handle this specific unsolicited finding is still ongoing and that no (international) consensus exists [Bombard and Miller, 2012; Bombard et al., 2012; Ross, 2012]. In this example, the result is anticipated and it concerns only one type of outcome (heterozygous carrier status of a specified recessive disorder), which makes the informed consent procedure less complex than with NGS in diagnostics.

### Feedback of Actionable Results from Genetic Research

In research, it is common practice that extensive informed consent is requested, whereas traditionally, there is no (or little) feedback of results. Increasingly, actionable results are being fed back to research participants, for example, in some population studies where new genomic techniques are used, with implications for the informed consent procedure [Knoppers et al., 2013]. An example comes from a cancer genetics study in Helsinki. This study, which started in 2011, uses NGS techniques, but unsolicited findings occur very rarely because of the targeted analysis. Research participants are given the option to be informed of any relevant finding from this study (Supp. Fig. S2).

Concerns are expressed about the practicalities inherent in feeding back research results including the validation of abnormal results and the implications for cascading clinically significant results when reporting findings [Miller et al., 2012]. These cascading obligations involve ensuring reliable and updated information and providing access to appropriate care that are often outside the scope of the research project, blurring the boundaries between research and clinical care [Hastings et al., 2012], and requiring specialist clinical and technical expertise.

### Changing Informed Consent for ArrayCGH

The use of ArrayCGH is an example from diagnostics where unsolicited findings are anticipated. With this technique, chromosomal aberrations in the form of copy number variations can be detected by cohybridizing sample and control DNA strands, looking for gains (duplications) and losses (deletions) of nucleotides in specific regions of the DNA [Albertson and Pinkel, 2003]. One experience of this early “genotype first” approach [Mefford, 2009] in diagnostics has been described by Schwarzbraun et al. (2009), where ArrayCGH was used to diagnose a 7-year-old boy with severe visual impairment, muscle hypotonia, psychomotor retardation, and seizures. The analysis showed the boy had a deletion of the *p53* gene, increasing the risk for a Li-Fraumeni syndrome, resulting in a 50% cancer risk in the first three decades of life [Schneider and Garber, 1993]. Netzer et al. (2009) reported a similar case with using ArrayCGH, after which they changed their informed consent procedure to expressly decide whether: (1) patients wish to be informed about any additional genetic finding with predictive value for the health of the proband and potentially his/her family; (2) they only wish to be informed about such additional genetic findings if effective treatment options or surveillance programs are available; or (3) they wish to be informed about carrier status for an autosomal recessive disease [Netzer et al., 2009].

Others have also categorized findings to enable patients to make a well-informed decision about having the test or not, without options for different feedback policies (e.g., see example of prenatal ArrayCGH at the VU University Medical Center, Supp. Fig. S3). Because of complex normative issues (including in some cases decisions about termination of pregnancy) with prenatal testing, it was perceived even more relevant to prepare parents for the possible outcomes by presenting different categories of potential findings in the pretest counseling.

### Consent for Unsolicited Findings from NGS in Diagnostics

NGS is increasingly being used in the clinical setting. An example of an informed consent form for exome sequencing in diagnostics at the Radboud University Nijmegen Medical Centre (Nijmegen, The Netherlands) can be found in Supp. Figure S4. Here, the patient (or his/her representative) consents to the targeted as well as the possibility of subsequently less targeted analysis of the sequence. In this example, it was decided that an Advisory Board (in the form described as an independent committee), consisting of a group of independent experts from different relevant disciplines, should decide whether an unsolicited finding should be reported to the referring medical doctor. The patient (or his/her representative) therefore does not agree to specified categories of information to be fed back, and there is no opt out possibility for feedback of unsolicited findings: if a patient does not want to be informed about relevant unsolicited findings, he/she is not eligible for the test.

This solution was developed because with the current experience it was perceived difficult to predict how many and which unsolicited findings could be expected, making it impossible to inform the patient about this. Furthermore, the possible conflict between a patient's opt out for unsolicited findings and the physicians’ duty to inform was considered to be difficult to explain to patients. Together, this justified the, possibly temporary, solution of not offering an opt out option for unsolicited findings. In the mean time, experience is built on the risk of unsolicited findings [de Ligt et al., 2012] and the experiences of patients and physicians.

In summary, the examples described here show that each context led to a different focus for the discussion on the procedure for informed consent, which to a large extent is dependent on the decisions regarding the feedback policy. The arguments used in the decision processes can be used as input for developing an optimal procedure for the informed consent for NGS in diagnostics.

## Practical Needs and Considerations

From the expert meeting, it also became clear that there are several practical difficulties to overcome for the informed consent procedure for NGS in clinical diagnostics. The most prominent questions and needs are described below.

### Handling Results After Analysis

When discussing feedback of test results, it is not always clear what the term “result” entails. Is this the raw data, all variants discovered, or only the interpretable and communicated results? The default might be to refrain from disclosing raw sequence data, which have been gained in a clinical setting, based on the fact that it lacks obvious clinical utility. A pragmatic approach might be to only include data that were communicated to the patient in his/her records, but jurisdictions differ in the legal rights given to patients to obtain access to these records. There is also ongoing discussion on future use of the sequence and the original sample and because this poses other complex issues, for example, related to privacy and confidentiality [McGuire et al., 2008], a clear protocol for storage and secondary use should be in place, of which the patient should be informed and for which consent should be given [Porteri and Borry, 2008].

### Blurring of Boundaries Between Research and Diagnostics: Managing Expectations

If the same professionals are involved in both clinical care and research and as similar methods and techniques are used, it is not always easy to make the distinction between clinical diagnosis and research. In the case of NGS, the need to look beyond known causes of a disease implicates a method that resembles research practice more than the current diagnostic procedures. Because expectations of patients could differ between different contexts, it is important that the primary aim of the test is communicated. A way of distinguishing between clinical diagnosis and research might be to question whether the primary goal of testing is for individual patient benefit or rather for some wider generalizable purpose, instead of simply considering the source of the funding for sequencing. In general, it should also be clear from the Research Ethics Committee application and decision how the sample may be used for future research and what information should be provided to the patient. This is important also for primarily clinical samples as we can expect that the rapid technical development will increase the interest in continuing the clinical investigation in a research setting [van El et al., 2013].

### Cooperation and Communication Between the Different Parties Involved in NGS

**Figure 1:**
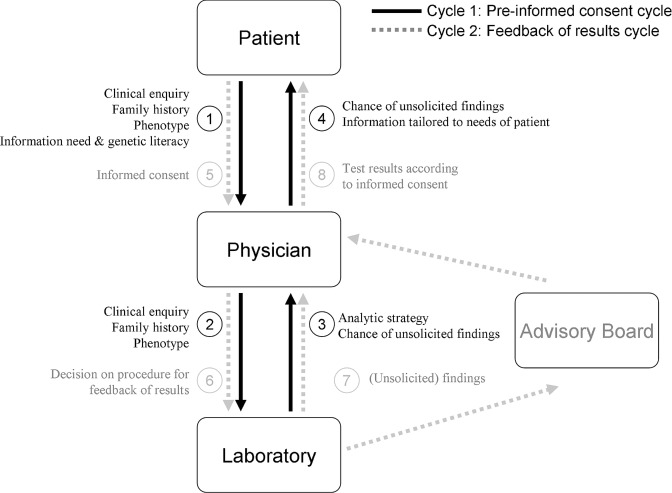
Cooperation and communication between different parties involved in the informed consent procedure for NGS in diagnostics.

To inform patients about what they are consenting to and to cater to the needs of different patients in various contexts, close cooperation between the different parties is required. Figure 1 depicts a model of the steps that should preferably be undertaken in the process of informed consent for unsolicited findings for NGS, assuming that consent is given ultimately to the full analyzing process (targeted and if needed broader). Ideally, the informed consent process in this case entails two “cycles of communication” (see Fig. 1). In the first cycle (or the preinformed consent cycle), the patient communicates the clinical enquiry together with relevant phenotypic characteristics and family history to the physician, whereas the physician assesses the patients’ needs and his/her genetic literacy (step 1). Preferably, the physician communicates the relevant information to the laboratory (step 2) to decide on the analytic strategy and the consequential risk of unsolicited findings. This information is passed to the patient by the clinician, tailored to the needs of the patient (step 3 and 4). In the second cycle, the patient gives informed consent to the scope of the results that are likely to be generated and a general strategy for feedback of results (step 5), which is communicated to the lab (step 6). The analysis and feedback of results (step 7 and 8) is executed accordingly, taking into account the needs of the patients as well as the interpretation of the outcome, both depending on the context (e.g., age of patient, reproductive future, family history, etc.) [Kaphingst et al., 2012].

It should be noted that this process depicts the ideal situation, optimizing information exchange between the different parties at the most appropriate time in the process. In practice, some of the steps in the cycles could be undertaken simultaneously (e.g., step 2 and 3) and might increasingly be automated, to speed up the process [Wright et al., 2011]. Similarly, a primary consent to targeted analysis (similar to other genetic tests) and a separate consent after ineffective targeted analysis are conceivable.

### A Changing Role for the Lab

With the introduction of NGS in diagnostics, the role of the laboratory geneticist changes. Detailed information on the phenotype and other patient characteristics are now essential to decide on the procedure to follow for analyzing the genotype [Gilissen et al., 2012; Hennekam and Biesecker, 2012]. This requires that the clinical geneticist and the laboratory geneticist become more attuned early in the diagnostic process, as depicted in Figure 1, which has implications for communication, training, and education of both specialists.

When a targeted approach is expected to be successful, this might yield fewer unsolicited findings, thus the counseling of the clinical geneticist should include information from the laboratory about the amount and type of expected unsolicited findings. Although the developing consensus in Europe is, where possible, to avoid finding unsolicited disease-related variants [van El et al., 2013], some others argue that particular known disease causing variants should be actively sought for and followed up when performing NGS in diagnostics [Green et al., 2013]. Besides the practical difficulties, this could amount to screening for conditions that would not pass current requirements for the initiation of screening programs [Andermann et al., 2008]. Moreover, it is questionable whether it does justice to the patients’ right not to know when patients are denied of the test if they decline to receive these variants.

The need to respect the patients’ right not to know also has implications for the analytic strategies. In general, it is argued that generating findings that are known to be clinically actionable and not related to the clinical enquiry should be avoided as much as possible [van El et al., 2013]. However, it might not always be feasible to specifically block such variants when analyzing the sequence, because it could include variants relevant for the diagnostic enquiry, and furthermore, it will be challenging to keep an updated list of all known disease-related variants.

### Which Unsolicited Findings Should be Communicated?

To aid communication and explanation about types of unsolicited findings and to facilitate patient decision making in the context of informed consent, several attempts have been made to categorize possible unsolicited findings, sometimes referred to as “binning” [Berg et al., 2011; Bredenoord et al., 2011; Vermeesch et al., 2012]. Categories can be defined by the nature of the condition, differentiating between early and late-onset diseases, the level of risk/predictivity, burden of the disease/severity, and options for treatment or prevention.

Given the physicians duty of care and taking account of what has been said about the patient's right not to know (see above), the default position should be that unsolicited findings revealing diseases or disease risks that require immediate medical treatment or prevention must be disclosed [Berg et al., 2011; Committee of Ministers and the Parliamentary Assembly of the Council of Europe, 2008]. This category should in any case be clearly defined and communicated prior to testing. Opting out or consenting to feedback of other categories was suggested by different professionals attending the expert meeting in March 2012, Amsterdam. An attempt to categorize different types of results from NGS in diagnostics according to their perceived need to feedback is depicted in Supp. Table S2. For some findings, it is very clear, and consensus was easily reached on, for example, never to disclose variants of unknown significance. Other findings (e.g., untreatable late-onset disorders) however raised more discussion and no final consensus was reached on the different categories, as was also experienced by others [Lohn et al., 2013]. The complexity of defining the results that should (or should not be) communicated lies mainly in the fact that this is dependent on the context (e.g., whether reproductive decision making is still relevant and if there is a family history of genetic disorders). Furthermore, the categories might differ between adults and children, because different ethical challenges might be raised for children [Bredenoord et al., 2013; Dondorp et al., 2012; Lantos et al., 2011].

### Limits to Informing and Decision Making

In general, it is very difficult (if not impossible) to inform patients about all possible kinds of outcomes. Certain groups of patients (e.g., less well-educated people and people from minority racial/ethnic groups) may have even more difficulty with processing the information from the informed consent procedure for genome sequencing and therefore require special attention [Kaphingst et al., 2012]. Currently, most patients considered for exome sequencing are children or cognitively impaired people. In these cases, proxy consent is usually taken, as the patient lacks the capacity to consent for himself/herself. Despite the inherent difficulties with providing sufficient information and with facilitating the decision-making process, our view is that in principle patients should have the right to opt out from feedback of unsolicited findings, because they fall outside the primary aim of the test.

It is anticipated that in the future it will not only be clinical geneticists, but also other specialists that will be involved in the informed consent process for NGS. The participants of the 2012 expert meeting concluded that in the short term, and until clear guidelines have been developed, it is preferred that a clinical geneticist is involved at least when disclosing results, because of his/her expertise in dealing with complex and sensitive genetic issues. In the medium term, it seems likely that clear guidelines will be developed based on current experiences that might facilitate the involvement of other appropriately qualified health professionals in communicating NGS findings to patients. For example, more debate is required to reach consensus on the exact elements of information required for informed consent [Ayuso et al., 2013].

### Involvement of an Advisory Board for Unsolicited Findings

In the transitional phase, while gaining more experience with unsolicited findings with diagnostic NGS, it may be helpful to involve an independent Advisory Board in the decision on follow-up of unsolicited findings, in particular when it is considered difficult to decide what to disclose. For this procedure, according to the participants at the 2012 expert meeting, the following points should be considered:

1. The results should be confirmed (with traditional methods, for example, Sanger sequencing) before they are communicated to the Advisory Board.
2. The Advisory Board should consist of individuals from different disciplines, preferably including a laboratory geneticist, a clinical geneticist (if possible independent of the case discussed), an ethicist, a legal expert (trained/experienced in the subject of NGS and unsolicited findings), and preferably a lay member/social worker (to represent the patients perspective).
3. It should be clear that the Advisory Board only has an advisory role: the clinical geneticist treating the patient is responsible for the final actions undertaken.
4. It is preferred that the treating clinical geneticist is informed about the result only after the Advisory Board has given its advice to disclose the findings, to protect the clinician from an internal conflict between the patient's right not to know and the duty to inform.
5. While in the beginning more cases will be discussed by the committee, it might be most practical that (local) standards are developed as soon as possible for more common outcomes, and the Advisory Board will only be consulted for more difficult cases.

### Conclusions

The development of NGS technologies, and their use in clinical settings for diagnosis, arguably offers new challenges to the feasibility of the requirement for informed consent as traditionally understood. One of the main conflicts in NGS for diagnostics is encountered between the “right to know” and the “right not to know” of the patient and his/her family on the one hand and the duty of care of the clinician on the other. This becomes complex especially when proxy consent is given by a parent or caretaker of a minor. Traditionally, in clinical practice, the patient's autonomy is valued highly, but in the case of NGS, it is unclear whether it is feasible to help the patient make a fully informed decision. Although models for categorizing different types of results (“binning”) have been developed, there are some challenges to implementing these approaches. For example, it seems currently impossible for the lab to confirm every result when a patient decides he/she would like to be informed about all findings. Furthermore, even with a binning system, it remains difficult for clinicians to explain the complexity of all possible outcomes. Due to this complexity, even when comprehending all information provided, it might be very difficult for individuals to foresee the implications and decide on the various different feedback procedures beforehand. Although it may seem paternalistic and unlike the established procedures in clinical genetics, it can be argued that the final decision of what to feedback to the patient should be in the hands of the physician. The reason to choose this position is first, that prior to testing only general information can be discussed, whereas after the test results are available, professionals will have to decide about disclosure on the basis of specific outcomes; and second, that the possible health interests of the patient's relatives may be at stake.

To ensure an optimal effort to provide good practice in the informed consent procedure for NGS in diagnostics, cooperation and communication between different parties are needed in multiple steps in the process (see Fig. 1). Furthermore, we propose a set of questions to consider for systematically gaining insight in the degree of feedback the patient should and could be able to decide upon when reporting back results from NGS in diagnostics (see Box 1).

### Box 1. Points to Consider when Contemplating the Mode of Informed Consent for NGS in Diagnostics

1. Who is giving consent: the patient or his/her legal representative? (i.e., how strong is the “right not to know”)
2. What is the initial clinical enquiry?
3. Which unsolicited findings can be expected?
4. How can the different possible unsolicited findings be categorized? (Supp. Table S1)
5. What should be communicated to the patient?
  - Which pretest information? (Fig. 1: cycle of communication 1)
  - Which results? (Fig. 1: cycle of communication 2)
6. What does this mean for the consent procedure?
  - General/detailed?
  - Oral/written consent?
  - Advisory Board involved?
  - Opt in or opt out of unsolicited findings?

## 

By addressing each question, different facets of the informed consent procedure receive attention, ensuring an optimal effort to provide good practice in the clinical genetic service of using NGS in diagnostics.

With regard to best practice for informed consent for NGS, we are aware that this article cannot provide definitive policy recommendations, as this field is evolving rapidly, but hope that the systematic process set out in this article provides a sound basis for developing the informed consent procedure for NGS in diagnostics.

## Supporting Information

Disclaimer: Supplementary materials have been peer-reviewed but not copyedited.

Figure S1 . Blood spot card from the Netherlands (2009, simplified and translated). Downloaded WIREs website at: October 8, 2012. **Figure S2**. Informed consent form Tumor Genetic Research, Helsinki (2012, translated) Courtesy: Prof. Lauri Aaltonen. **Figure S3**. Consent statement disclosure of outcome of prenatal Array-CGH, VUMC Amsterdam, the Netherlands* (2011, translated). Courtesy: Kyra Stuurman. **Figure S4**. Consent form exome sequencing, RUNMC Nijmegen, the Netherlands (2011). Courtesy: Helger Yntema. **Table S1**. Workshop Amsterdam, March 21, 2012: Informed Consent for Exome Sequencing in Diagnostics **Table S2**. Proposed categories of unsolicited findings: which unsolicited results should be communicated?
