# Supplementary material for: Reflecting on Earlier Experiences with Unsolicited Findings: Points to Consider for Next-Generation Sequencing and Informed Consent in Diagnostics
Source: Hum Mutat. 2013 Jul 16;34(10):1322–8. doi: 10.1002/humu.22370 (PMC4285964; doi:10.1002/humu.22370)
Supplement: Figure S1 — . Blood spot card from the Netherlands (2009, simplified and translated). Downloaded WIREs website at: October 8, 2012. Figure S2. Informed consent form Tumor Genetic Research, Helsinki (2012, translated) Courtesy: Prof. Lauri Aaltonen. Figure S3. Consent statement disclosure of outcome of prenatal Array-CGH, VUMC Amsterdam, the Netherlands* (2011, translated). Courtesy: Kyra Stuurman. Figure S4. Consent form exome sequencing, RUNMC Nijmegen, the Netherlands (2011). Courtesy: Helger Yntema. Table S1. Workshop Amsterdam, March 21, 2012: Informed Consent for Exome Sequencing in Diagnostics Table S2. Proposed categories of unsolicited findings: which unsolicited results should be communicated? [file humu0034-1322-sd1.pdf]

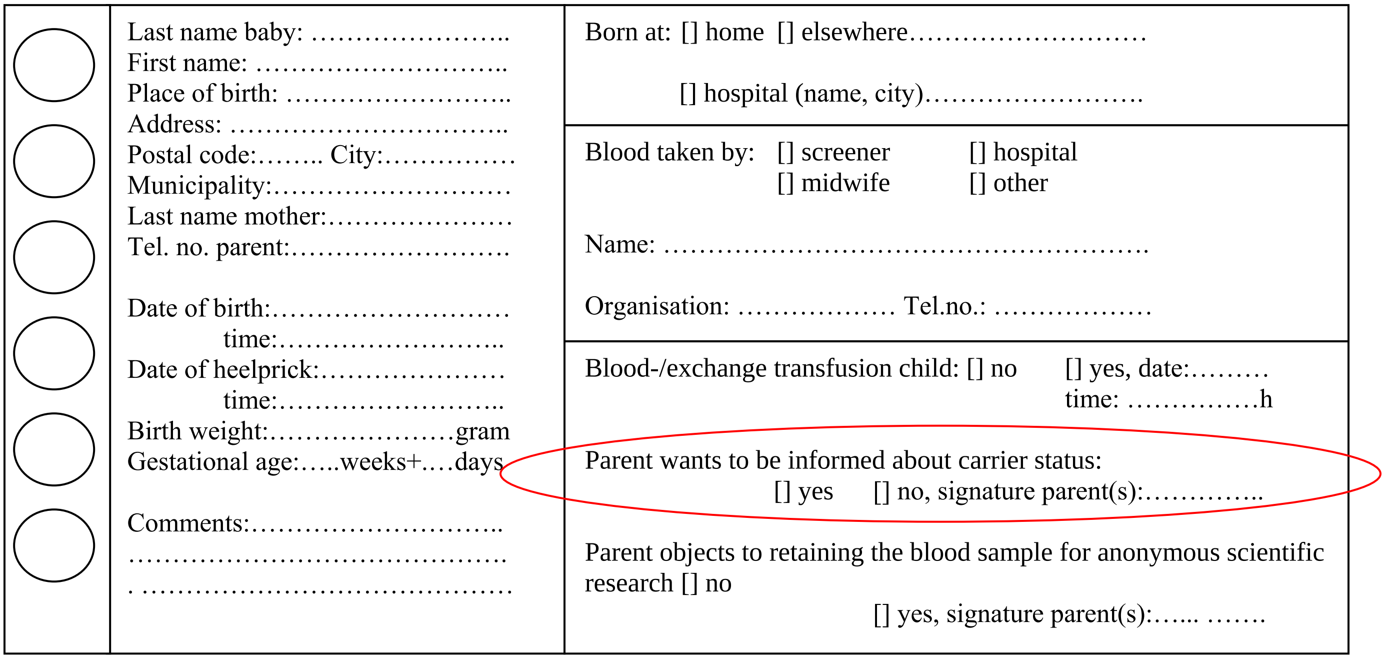

Last name baby: …………………..
First name: ………………………..
Place of birth: ……………………..
Address: …………………………..
Postal code:…….. City:……………
Municipality:………………………
Last name mother:…………………
Tel. no. parent:……………………

Date of birth:………………………
time:……………………..
Date of heelprick:…………………
time:……………………..
Birth weight:…………………gram
Gestational age:…..weeks+.…days

Comments:………………………..
……………………………………
. ……………………………………

Born at: [] home [] elsewhere………………………
[] hospital (name, city)……………………

Blood taken by: [] screener [] hospital
[] midwife [] other

Name: ……………………………………………….

Organisation: ……………… Tel.no.: ………………

Blood-/exchange transfusion child: [] no [] yes, date:………
time: ……………h

Parent wants to be informed about carrier status:
[] yes [] no, signature parent(s):…………..

Parent objects to retaining the blood sample for anonymous scientific research [] no
[] yes, signature parent(s):…... …….

**Supp. Figure S1.** Blood spot card from the Netherlands (2009, simplified and translated). Downloaded at: http://www.rivm.nl/ October 8, 2012.

I have read the letter outlining the tumor gene research. I understand that you want to take a blood / tissue sample from me and that the sample will be sent to a research laboratory for a molecular genetic study. The samples will be examined for changes in the genes that might contribute to tumor genesis.

I know that sampling is completely voluntary. I am free to participate in the study. I also know that I can withdraw from the investigation without notifying the cause and that I have the right to deny that the samples are analyzed if I would desire so. To participate in the study or to withdraw from it does not affect my potential therapies.

I understand that the study does not benefit me personally and I can not expect personal outcomes.

I understand that, although the method enables looking at the entire genome, the aim of the study is not to make a complete interpretation of my genetic information, but will focus on tumor genes. It is possible that changes are found that could be very important for my health and for which there are preventive options.

In this case (check the desired box):

o I wish to be contacted only about my genetic risk for tumor development
o I wish to be contacted about any finding that may be relevant for my health and for which there is a preventive measure available. I understand that many people are carriers of some hidden hereditary disease and that if such a genetic carrier status is found this will not be communicated.
o I do not want to be contacted, I am giving my sample purely to promote scientific research.

I give my consent to take samples for tumor genomic research, as well as giving permission to collect the information that is relevant to the investigation.

**Supp. Figure S2.** Informed consent form Tumor Genetic Research, Helsinki (2012, translated) Courtesy: Prof. Lauri Aaltonen.

With this declaration I, parent of my unborn child, state that I have been informed about the potential outcome of studies with array-CGH.

I agree with the following disclosure policy:

Every outcome relevant to the initial enquiry will be disclosed.

In the event that there is an outcome that is not related to the initial enquiry, but has health implications, the following applies (in practice the circumstances described below will occur very rarely):
1. If there is an outcome not related to the initial enquiry, but with direct consequences for the health of my unborn child, this will be disclosed
2. If there is an outcome without direct consequences for the health of my unborn child, but with consequences for a potential next pregnancy (carrier status for a recessive condition), this will be disclosed
3. If there is an outcome which relates to a condition occurring later in the life of my unborn child and for which treatment is available, this will be disclosed
4. If there is an outcome which relates to a condition occurring later in the life of my unborn child and for which no treatment is available, this will not be disclosed

**Supp. Figure S3.** Consent statement disclosure of outcome of prenatal Array-CGH, VUMC Amsterdam, the Netherlands* (2011, translated). Courtesy: Kyra Stuurman.

*This test is offered after multiple structural anomalies have been detected in the fetus with prenatal ultrasound. Untreatable late-onset disorders (although never found so far) were explicitly mentioned not to be disclosed, because they were perceived not to be actionable. Although this last category might be of personal utility to the child at a later age, the right not to know of the unborn child was used as the main argument not to disclose this to the parents, together with the perceived lack of urgency in this context.

1. I wish that DNA from **me/ my child / person under my legal guardianship** will be stored and tested by exome sequencing for the following condition: ____________________________________________

2. I understand that only genes known to cause the condition in question will be analyzed initially (*NB* all genes will be analyzed initially for some specific conditions). The test results will be discussed with me on completion of the analysis. If this initial testing does not identify a cause, all genes will then be analyzed (exome sequencing). I will also be informed if findings relevant to the condition in question are identified by exome sequencing.

3. I understand that there is a small chance that co-incidental findings not related to the specific condition in question may be identified.
I understand that any co-incidental findings will be assessed by an independent expert committee. Such co-incidental findings may be of medical importance. If so, the committee in consultation with my doctor, may inform me of these co-incidental findings. This will occur only if the co-incidental findings could have a significant impact on my health / the health of my child.

4. I understand that the knowledge of genetic conditions is likely to improve in the future. I would like to be contacted if further information becomes available about the exome sequencing results relating to the genetic condition in my family.

5. I understand that the information from the exome sequencing will be stored at the Department of Human Genetics, UMC St. Radboud and that it may be shared with researchers in other departments after it is de-identified.

6. I understand that I have the ability to withdraw my consent at any time without influencing **my management /the management of my child/my ward.**

7. I have had the opportunity to ask additional questions and I am satisfied with the explanations.

**Supp. Figure S4.** Consent form exome sequencing, RUNMC Nijmegen, the Netherlands (2011). Courtesy: Helger Yntema.

**Supp. Table S1. Workshop Amsterdam, March 21, 2012: Informed Consent for Exome Sequencing in Diagnostics**

EuroGentest

WP8: Best Practice Guidelines for Provision of Clinical Genetic Service

**Workshop: Informed Consent for Exome Sequencing in Diagnostics**
**March 21, 2012**

Technology in genetic health care services is developing at a high speed, exerting in a major impact on the provision of health care services. Examples include shifts from curative to preventive services, from in-patient to out-patient treatment and from specialized genetic services to genetics as an integral part of general health services. In 2011, an international expert meeting entitled "Building excellence in clinical genetic services" was organized by Eurogentest2 in Zaandam, the Netherlands, funded by the European Commission (www.eurogentest.org), to identify topics within genetic service provision that need (more) guidance. The first priority identified was the need for best practice guidelines for an informed consent procedure to be used when implementing next generation sequencing in clinical diagnostics. To share experiences and further explore dilemmas, a subsequent workshop has been organized in Amsterdam on March 21, 2012:

*Workshop Part 1: Experiences so far*
Exchange of experiences from practice and literature

*Workshop Part 2: What can we learn?*
Discussion on possible implementation of "binning system" and implications for informed consent procedures

Participants:

| **Name** | **First name** | **Country** | **Professional background** |
|---|---|---|---|
| *Organisation:* | | | |
| Cornel | Martina | NL | Community Genetics and Public Health Genomics |
| Henneman | Lidewij | NL | Health Sciences |
| Kristoffersson | Ulf | SE | Clinical Genetics |
| Rigter | Tessel | NL | Research |
| van Aart | Charlotte | NL | Student Health Sciences |
| *Participants:* | | | |
| Borry | Pascal | BE | Ethics |
| Tönnies | Holger | DE | Clinical Molecular Genetics |
| Kranendonk | Elcke | NL | Jurist/research |
| Ijntema | Helger | NL | Clinical Molecular Genetics |
| Hall | Alison | UK | Policy advisor PHG foundation |
| Dondorp | Wybo | NL | Ethics |
| Ploem | Corrette | NL | Jurist |
| Waisfisz | Quinten | NL | Clinical Molecular Genetics |
| Elting | Mariet | NL | Clinical Genetics |

**Supp. Table S2. Proposed categories of unsolicited findings: which unsolicited results should be communicated?**

| **Always**[a] | **If consented** | **Never** |
|---|---|---|
| - Findings indicating an emergency situation | - Validated & clinically relevant findings (medical actionable)<br>(e.g. carrier status of autosomal recessive disease) | - Variants of unknown significance<br>- Findings with low predictive value<br>- Findings with low/no clinical utility |
| *Depending on context:*<br>*- When the patient is a minor: All actionable, treatable and serious conditions*[b]<br>*- When the finding is of clinical relevance for family member(s)* | *Depending on context:*<br>*- Life style related variants (moderate clinical utility)*<br>*(e.g. alpha-1-antitrypsin deficiency*[c]*)* | *Depending on context:*<br>*- Untreatable late onset disorders*[d]<br>*- Just for personal curiosity*[e] |

A proposal made by participants at the expert meeting in Amsterdam, March 2012.

The types of results under "Depending on context" could be either decided before start of NGS by the patient or legal representative or by the clinician (in some cases with assistance from an independent Advisory Board).

[a] This seems to be the most difficult category to define (what defines an "emergency situation?"), partly because it might not be generalizable. This category should be clearly defined and communicated prior to testing, because the patient should be aware that with regard to this category he/she cannot claim an absolute 'right not to know'.
[b] Possibly depending e.g. on age of patient and whether it is feasible to decide on the information at a later age him-/herself.
[c] It was argued that this information in some cases could lead to life-style adjustments (e.g. non-smoking) that could prevent serious health problems.
[d] Exceptions are possible here; might be relevant in some cases.
[e] It was argued that it will currently be unmanageable and too costly to confirm these types of results.
